# Acute appendicitis: echographic diagnosis at Emergency Departament

**DOI:** 10.1186/2036-7902-4-S1-A27

**Published:** 2012-12-18

**Authors:** Alberto Oviedo-García, M Algaba-Montes, J Lopez-Libano, JM Alvarez-Franco, N Diaz-Rodriguez, A Rodriguez-Lorenzo

**Affiliations:** 1 Emergency Department, Valme Hospital, Seville, Members of the Working Group of ultrasound SEMES_Andalucía and Semergen, Spain; 2 Critical Care Department, Miramar Hospital, Mallorca, Member of the Working Group of ultrasound Semergen, Spain; 3 Emergency Department, IB-Salut, Ibiza, Member of the Working Group of ultrasound, Semergen, Spain; 4 Primary Care, Barbadás Primary Care Center, Ourense, Member of the Working Group of ultrasound Semergen, Spain; 5 Radiology Department, Perpetuo Socorro Hospital, Vigo, Member of the Working Group of ultrasound Semergen, Spain

## Background

Acute appendicitis (AA) is the most frequent abdominal emergency surgery and the perforation, is mainly due to a delay in the diagnosis. The use of ultrasound (US) in Emergency Department (ED), could avoid delays in the diagnosis of this entity. When there is a perforation surgical morbidity multiplies by 15 and 50 the deaths.

## Objective

We present a case of AA, diagnosed at ED, through the use of US scanning used by Emergency Physicians (EP), and promoting their use.

## Patients and methods

A patient with abdominal pain, with a final diagnosis of an AA assessing ultrasound, performed by EP. We used a Sonosite M Turbo, equipped with probe Convex C60 between 2 and 5 MHz.

## Results

A male patient 18 years old, who attends the ED services, with abdominal pain, located all along his right side. No sign of nausea, vomiting or diarrhea, no fever or dysuria. He came in walking in the surgery, conscious and lucid, well hydrated and perfused, afebrile; abdominal tenderness presented an abdomen with voluntary defense in right hemiabdomen, no sign of peritoneal irritation. The rest of the exploration was normal. We have a slight leukocytosis of 10,900 without findings anywhere else in complementary tests. The patient continued with the same pain and the abdominal condition had not changed, so an abdominal US was performed, discovering an enlarged appendix, absence of peristalsis, not compressible, and the thickened wall.

## Conclusion

Ultrasound carried out by EP, can be a very useful tool in cases for which clinic and analytics are not clear. The sensitivity of US for the diagnosis of AA is high, vary from 80 to 94%, but is highly browser dependent and it is essential therefore, to have an appropriate training of the MU, to prevent diagnostic errors. To incorporate the US in ED decreases overall care time, since the EP is more efficient and dynamic, providing greater clinical safety and decreasing the complications.
